# Comparison of lower limb lifting and squeeze exsanguination before tourniquet inflation during total knee arthroplasty

**DOI:** 10.1186/s12891-019-2406-6

**Published:** 2019-01-22

**Authors:** Meng Zhang, Gang Liu, Zexue Zhao, Pengfei Wu, Weidong Liu

**Affiliations:** Department of Orthopedic Surgery, The Affiliated Huaian No.1 People’s Hospital of Nanjing Medical University, 6 Bejing Road West, Huai’an, 223300 Jiangsu Province China

**Keywords:** TKA, Tourniquet, Exsanguination, Skin tension blister, VAS score

## Abstract

**Background:**

During total knee arthroplasty(TKA), tourniquet is widely used by most surgeons whereas the optimal application is still controversial. With this prospective randomized controlled study, we intend to investigate the effect of lower limb lifting and squeeze exsanguination methods on clinical outcomes in a series of TKAs.

**Methods:**

Prospectively enrolled a total of 236 TKA patients from March, 2012 to November, 2016. Of which 118 patients randomly constitute Group A with lower limb lifting exsanguination technique; and the other 118 patients comprise Group B with squeeze exsanguination method. A year’s follow-up measurements were recorded in detail for analysis.

**Results:**

The pre-tourniquet time of Group A was significantly shorter than that in Group B (*P* < 0.001). Significant difference was found on skin tension blister, 3 happened in Group A and 11 happened in Group B (*P* = 0.031), which resulted in a difference in total complications (*P* = 0.039). The VAS score was significantly lower in Group A at one and seven days postoperatively, *P* < 0.001 and *P* = 0.011, respectively. No significant differences were found regarding all other clinical outcome measurements.

**Conclusion:**

The lower limb lifting exsanguination is a safe and effective technique. Compared with squeeze exsanguination method, it could decrease the incidence of skin tension blister and alleviate early postoperative pain reaction, no additional risks occurred regarding other clinical outcomes. Thus, it might have the potentiality to be commonly utilized in TKA procedure.

**Trial registration:**

ClinicalTrials.gov Identifier: ChiCTR1800020471. Registered on 31 December 2018 Retrospectively registered.

## Background

Total knee arthroplasty (TKA) has been a successful procedure for reducing pain and restoring function in cases with end-staged rheumatoid arthritis and osteoarthritis [1]. The tourniquet, since first proposed by Dr. Lister [2], was commonly used by most surgeons in orthopedic surgery. However, the use of a pneumatic tourniquet has long been debated with a growing attention especially in recent years [3–7]. Increasing studies have focused on the different strategies of the tourniquet use in regarding of use or not use [8] and how to optimally use. Techniques differentiated among studies, such as releasing tourniquet before wound closure [9, 10]; releasing it prior to dressing application [11]; releasing it after implantation of the prosthesis [12]; using tourniquet from osteotomy to wound closure [13] or only among the period of prosthesis implantation [14].

Meanwhile, different lower limb exsanguinations before tourniquet inflation have been reported, such as the Esmarch bandage, the Urias bag, the Northwick-park exsanguinator, Rhys-Davies mechanical exsanguinator, the lower limb lifting and the hand-over-hand squeeze exsanguination [15–18]. In order to identify an optimal exsanguination method, a few clinical studies have been conducted to compare the effect of different preoperative exsanguination methods on the clinical outcomes. Blond et.al [15] compared different exsanguination methods of the upper limb in healthy young volunteers, and concluded that the squeeze method was the best before inflation of a tourniquet; Tanpowpong et.al [17] found a significant lower tourniquet tolerance of exsanguination with a tight bandage compared to lifting method in the upper arm; Farbood et.al [16] focused on the Esmarch bandage and limb lifting of the upper extremity and found the limb lifting technique produced less discomfort for patients; Angadi et.al [18] investigated the use of Rhys-Davies mechanical exsanguination and limb lifting method in knee arthroscopy, and found that lower limb lifting was an effective technique of exsanguination prior to knee arthroscopy. These studies were mostly related to upper limb combined with various diversities and conflict conclusions, which limited the application of the clinical findings. Furthermore, as reported, some of these techniques require special cautions, especially in the presence of a clot in deep vein, infection, unstable fractures, malignancy and latex allergy [16, 18]. The Rhys-Davies exsanguinator has been reported to burst whilst being used for exsanguination [19]. And several studies reported fatal pulmonary embolism subsequent to the use of Esmarch badage and tourniquet [20–22].

The hand-over-hand squeeze exsanguination technique is a procedure on the lower limb starting with a squeezer from the foot, gradually proceeding and forcing the blood into proximal limb through the tourniquet [15, 23]. This may have a similarity with esmarch exsanguination and Rhys-Davies exsanguinator, which may also drive a clot in deep vein to cause a fatal pulmonary embolism. Furthermore, the asymmetrical pressure caused by squeezer may damage the soft tissue, resulting in pain and discomfort. Hence, hunting for an appropriate technique to avoid the potential risks appears to be necessary. As far as we know, the investigation of the effect of exsanguination techniques on clinical outcomes during TKA remains vacant. Therefore, we designed this prospective randomized controlled study to detect the clinical difference between the lower limb lifting and hand-over-hand squeeze exsanguination before tourniquet inflation during TKA.

## Methods

### Patients recruiting

The consolidated standards of reporting trials (CONSORT) statement was strictly followed as to conduct this prospective randomized controlled study. All patients who underwent unilateral primary TKA were initially considered from March, 2012 to November, 2016. Exclusive criteria included rheumatoid arthritis, tuberculous arthritis, traumatic osteoarthritis and a history of previous knee surgery; a series of comorbidities, including a history of anemia, dysfunction of coagulation, severe diabetes mellitus and hypertension without good control. The study was approved by the Human Research Ethics Committee of our hospital, and all the participants were completely informed and signed written consent forms according to the protocol.

### Operative technique and interventions

The sub-divisional departments of our hospital provided a standardized perioperative care for the patients undergoing TKA. All recruited participants underwent the operative procedure with the same general anesthesia. A standard TKA procedure was then performed through a perpendicular midline incision and a medial parapatellar approach. The peripheral osteophytes of the proximal tibia and the distal femur were initially removed, and then an intramedullary and extramedullary guidance was used for the femur and tibia osteotomy respectively. After accomplishment of the prosthesis implantation, the wound was closed after irrigation, hemostasis and cocktail (5 mg morphine, 30 mg bupivacaine and 1 ml betamethasone, mixed with sterile normal saline solution to a volume of 60 ml) local injection, one drainage tube was inserted. The operations were all performed by a senior surgeon in our hospital with the other three surgeons assisted. All the procedures were same except the preoperative exsanguination method. A posterior cruciate-substituting cemented prosthesis was used in every knee arthroplasty (GENESIS II,Smith & Nephew, Orthopaedics AG, Switzerland).

**Fig. 1:**
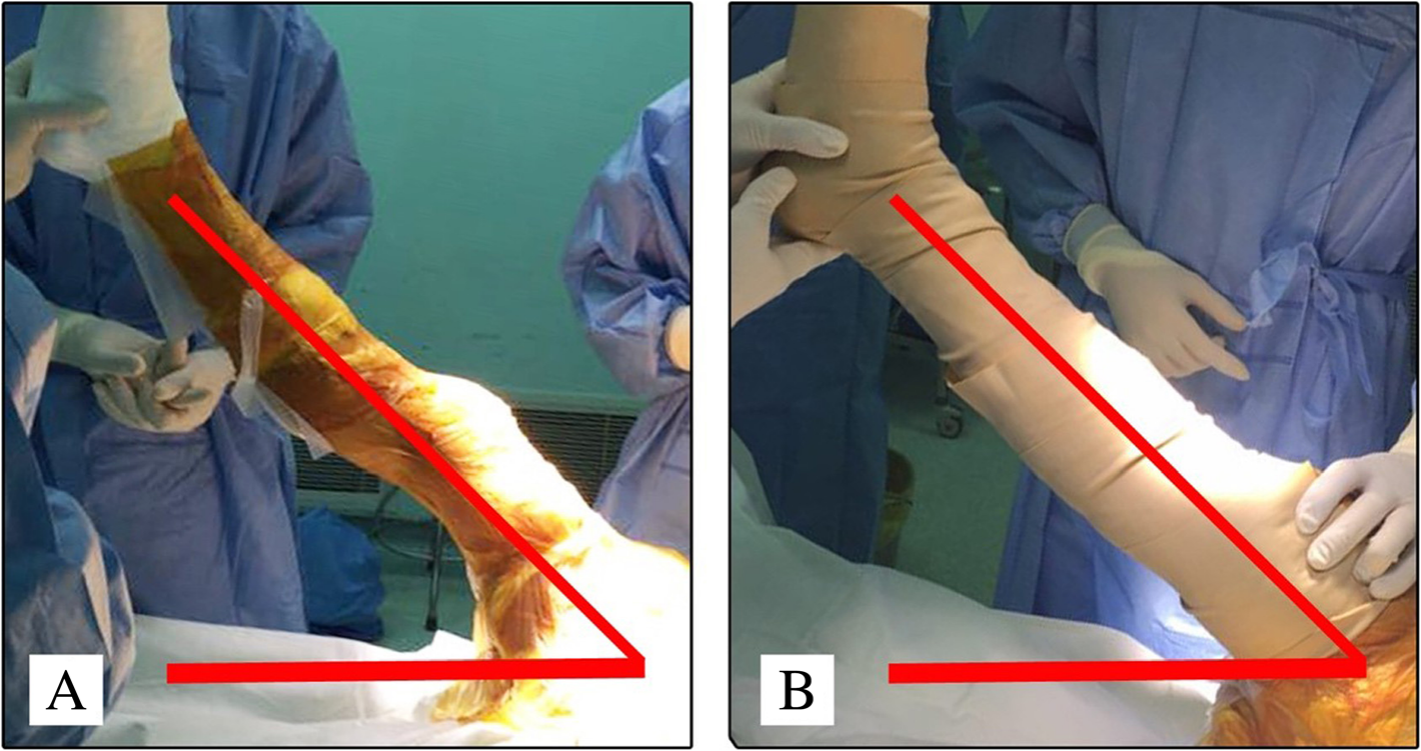
**a**: The leg elevation exsanguination method; **b**: The hand-over-hand squeeze exsanguination method

In the lower limb lifting group, after skin being sterilized and drapes being set up, the senior surgeon lifted the lower limb at 45 degrees and maintained for 30s, followed by inflation of the pneumatic tourniquet [15, 24]. In the hand-over-hand squeeze exsanguination group, the assistant surgeon lifted the limb at 45 degrees and maintained. The senior surgeon wrapped the lower leg with an elastic rubber squeeze under the protection of gauze pad. Of all the patients, the pneumatic tourniquet was fixed by a same surgeon. The pneumatic tourniquet was inflated to a pressure of systolic blood pressure plus 100 mmHg; and deflated right after the implantation of the prosthesis accomplishment. A photo of both procedures was shown as Fig. 1 for better understanding (Fig. 1).

An oral non-steroidal anti-inflammatory drug (Celecoxib Capsules, 200 mg, qd) was prescribed for pain control regularly postoperatively if no contraindication exists. Chemical thromboprophylaxis was not prescribed in this trial. Fluid supplement was standardized in both groups. All the patients were mobilized according to a standardized physical therapy protocol under the guidance of the doctors and nurses from the first postoperative day. Drainage was removed in 24 h. The criterion of a blood transfusion was set as a hemoglobin (Hb) level < 8 g/dL or < 10 g/dL with symptomatic anemia.

### Outcome measurements

All patients were followed 1 year postoperatively for inspection of complications, visual analogue scale (VAS) score and Range of Motion (ROM), HSS and 36-item short form survey (SF-36) scores. The primary clinical outcome was VAS score and complications including wound oozing, skin incision marginal necrosis, skin tension blister, wound hematoma, superficial and deep infection, and deep vein thrombosis (DVT) or pulmonary embolism (PE). The secondary outcomes were the tourniquet time, operation time, blood loss (intraoperative, postoperative drainage volume, calculated blood loss), knee functions (time of achieving 90° knee flexion and straight leg raise, ROM), HSS score and SF-36 score at 1 year postoperatively. The tourniquet time and operation time were recorded by an independent observer. The intraoperative blood loss was calculated by measuring the suction volume and weighing the gauze. Postoperative blood loss was considered as the drainage volume. Calculated blood loss was obtained by the method proposed by Gross [25]. The knee ROM was measured three times with a leg goniometer and the average value was recorded by an independent observer. The VAS measurement for pain was performed by placing a cross on a straight line with a 10 cm in length and the result was recorded from the left side in cm. The SF-36 score was constituted by physical component summary (PCS) and mental component summary (MCS), which has been widely used in the subjective evaluation of outcomes after TKA [26]. The HSS score and SF-36 score were all obtained by each patient using the questionnaire at 1 year postoperatively. Because a few patients were lost to follow during the year, we excluded them after the time point they were lost when conducting the analysis. However, previous existed data was still counted for analysis. Due to the complications could significantly influence the VAS score and functions, we also excluded those patients when conducted related analysis.

### Randomization and blinding

The randomization was generated by a computerized random sequence with a sealed envelope method, and the sequence was concealed until the intervention was assigned in the operation room. The demographic baseline data, intraoperative and postoperative clinical outcomes were collected by two independent observers. Observers and the patients were blinded to the allocation.

### Power analysis

The sample size was calculated based on the primary outcome (i.e. VAS score) to detect a VAS score’ difference of a 0.5 score. According to a previous study [27], the standard deviation of VAS score at 7 days postoperatively was 1.1, thus a total of 100 patients in each group were required to detect the difference with a 90% power and a single tail alpha of 0.05. A recruitment of nearly 120 patients were determined per group with a drop-out rate of 20%.

### Statistical methods

Data analysis was performed by using the standard statistical software (SPSS 19.0, Inc. USA). Categorical variables were presented as absolute number and relative frequencies. Chi-square test was used to test the differences. Continuous variables were presented as the Mean ± Standard deviation (SD). Mann-Whitney U test was used in nonparametric data and Student-t test was used to detect parametric data. Results were considered as significant if *P* < 0.05.

## Results

### Patients flow

**Fig. 2:**
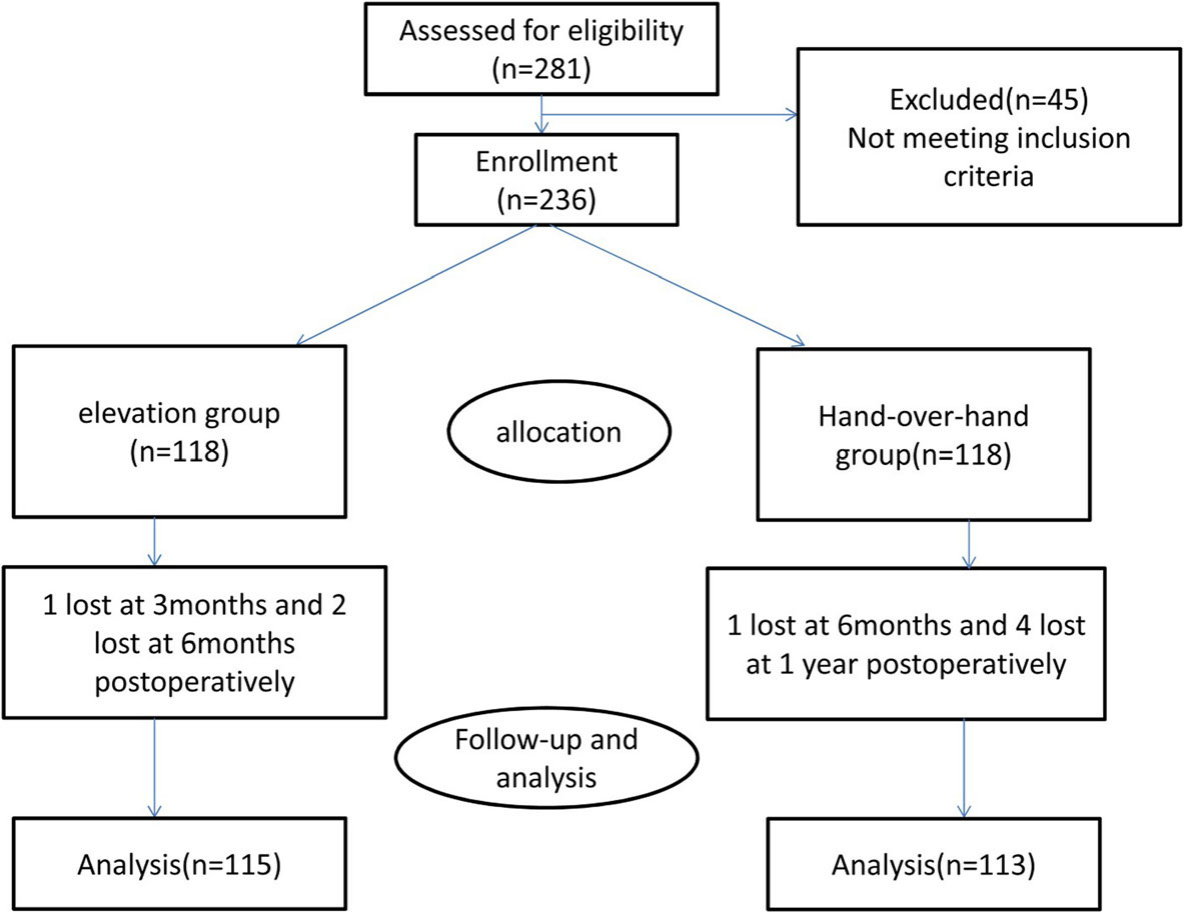
Patients’ flowchart

Two hundred eighty-one patients were initially enrolled. Twenty-seven patients were excluded based on the exclusive criteria and 18 declined to participate with a final of 236 patients recruiting for the trial. During the follow-up, 3 patients in Group A and 5 patients in Group B were lost to follow, leaving 115 patients in Group A and 113 patients in Group B into the final analysis. A CONSORT flow diagram was presented (Fig. 2).

### Demographic baseline data

The demographic data was equally matched between the groups regarding age, gender, Body Mass Index (BMI), preoperative Hb, VAS score, HSS score, SF-36 score and knee ROM. The baseline data was presented in Table 1.

**Table 1:** Preoperative baseline data

Parameters	Group A(*n* = 118)	Group B(n = 118)	*P* value
Age	68.4 ± 8.8	69.5 ± 9.2	0.513
Male/Female	48/70	52/64	0.521
BMI	24.9 ± 2.3	25.2 ± 2.4	0.773
HB(g/L)	129.8 ± 10.2	127.6 ± 9.8	0.614
VAS score	4.8 ± 1.3	4.6 ± 1.5	0.368
HSS score	56.8 ± 17.6	59.2 ± 16.9	0.254
ROM	118.2 ± 12.1	121.1 ± 11.7	0.472
SF-36(PCS)	45.2 ± 13.7	44.7 ± 14.1	0.334
SF-36(MCS)	46.3 ± 15.4	47.2 ± 14.8	0.671

*BMI* body mass index, *HB* hemoglobin, *VAS* visual analogue scale, *HSS* the hospital for special surgery, *ROM* range of motion, *SF-36(PCS)* 36-item short form survey (physical component summary), *SF-36(MCS)* 36-item short form survey (mental component summary)

### Clinical outcomes

Detailed perioperative data were presented in Tables 2, 3 and 4.

**Table 2:** Follow-up outcomes

Parameters	Group A	Group B	*P* value
Pre-tourniquet time(s)	40.7 ± 4.8	156.4 ± 10.7	< 0.001*
Tourniquet time(min)	48.2 ± 5.3	46.7 ± 5.1	0.312
Operation time(min)	71.6 ± 7.4	69.8 ± 8.2	0.668
Intraoperative blood loss(ml)	123.3 ± 22.1	114.7 ± 18.6	0.162
Postoperative drainage volume(ml)	281.6 ± 33.6	287.4 ± 31.7	0.771
Calculated blood loss(ml)	841.5 ± 118.3	816.3 ± 131.2	0.226
The time of achieving 90° flexion(days)	1.5 ± 0.3	1.6 ± 0.4	0.462
The time of achieving SLR(days)	1.9 ± 0.9	2.1 ± 1.1	0.172
HSS score	86.1 ± 14.3	88.2 ± 15.1	0.745
SF-36(PCS)	49.6 ± 9.6	50.3 ± 8.8	0.813
SF-36(MCS)	55.4 ± 10.3	53.1 ± 9.8	0.382

*HSS* the hospital for special surgery, *SLR* straight leg raise, *SF-36(PCS)* 36-item short form survey (physical component summary), *SF-36(MCS)* 36-item short form survey (mental component summary); * indicates a significant difference

**Table 3:** Complications

Parameters	Group A(*n* = 118)	Group B(*n* = 118)	*P* value
Oozing	3	2	0.651
Marginal necrosis	0	1	0.316
Skin tension blister	3	11	0.031*
Wound hematoma	3	5	0.472
DVT and PE	1	2	0.561
Superficial infection	1	0	0.316
Deep infection	0	1	0.316
Total complications	11 (9.3%)	22 (18.6%)	0.039*
Total complications exclude skin tension blister	8 (6.8%)	10 (8.5%)	0.624

*DVT* deep vein thrombosis, *PE* pulmonary embolism, * indicates a significant difference

**Table 4:** VAS score and ROM

Parameters	Group A	Group B	*P* value
VAS (PO 1d)	4.5 ± 1.0	5.3 ± 1.2	< 0.001*
VAS (PO 7d)	2.9 ± 0.8	3.5 ± 0.9	0.011*
VAS (PO 1 m)	1.5 ± 0.7	1.8 ± 0.8	0.116
VAS (PO 3 m)	1.2 ± 0.5	1.5 ± 0.7	0.212
VAS (PO 6 m)	0.9 ± 0.7	1.0 ± 0.8	0.663
VAS (PO 1y)	0.8 ± 0.6	0.8 ± 0.7	0.781
ROM (PO 7d)	109.4 ± 4.2	108.2 ± 4.8	0.463
ROM (PO 1 m)	113.1 ± 3.6	112.3 ± 4.2	0.618
ROM (PO 3 m)	120.4 ± 4.4	121.6 ± 5.1	0.364
ROM (PO 6 m)	122.1 ± 3.9	121.9 ± 4.3	0.572
ROM (PO 1y)	124.3 ± 4.8	123.7 ± 5.4	0.615

*VAS* visual analogue scale, *ROM* range of motion, *PO* postoperative, * indicates a significant difference

The pre-tourniquet time indicates from drapes being set up to the inflation of the tourniquet. It was 40.7 ± 4.8 s in Group A, which was significantly shorter than that in Group B (156.4 ± 10.7) seconds, (*P* < 0.001). No significant differences were found between the groups regarding tourniquet time and operation time, intraoperative blood loss, postoperative drainage volume and calculated blood loss.

Significantly, we found a decreased VAS score in the early postoperative days in Group A, at 1 day and 7 days postoperatively respectively (*P* < 0.05). Continuously to follow, at 1 month, 3 month, 6 month and 1 year postoperatively, no significant differences were found between the groups.

**Fig. 3:**
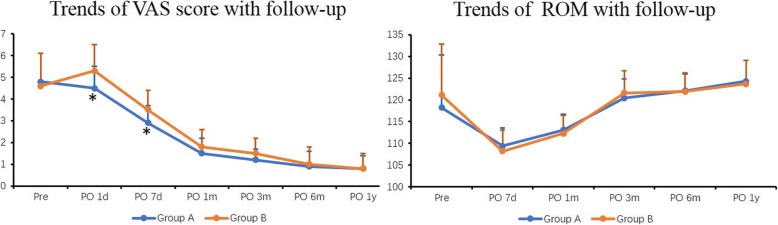
Trends of VAS and ROM with the follow-up time. * indicates a significant difference

Knee functions were recorded and analyzed. No significant differences were found on time of achieving 90° knee flexion and straight leg raise, and knee ROM during the follow-up. The HSS score, SF-36 PCS and MCS score improved compared with preoperative items, but no significant differences were found when compared between groups. Trends of VAS and ROM with follow-up time were presented as Fig. 3.

**Fig. 4:**
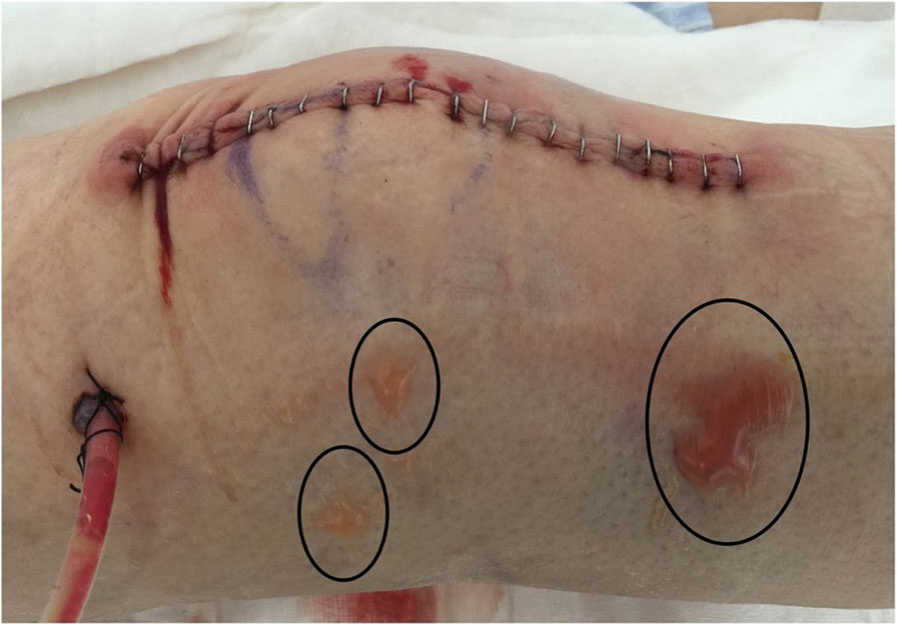
An example of the skin tension blister 1 day postoperatively (the circles)

**Fig. 5:**
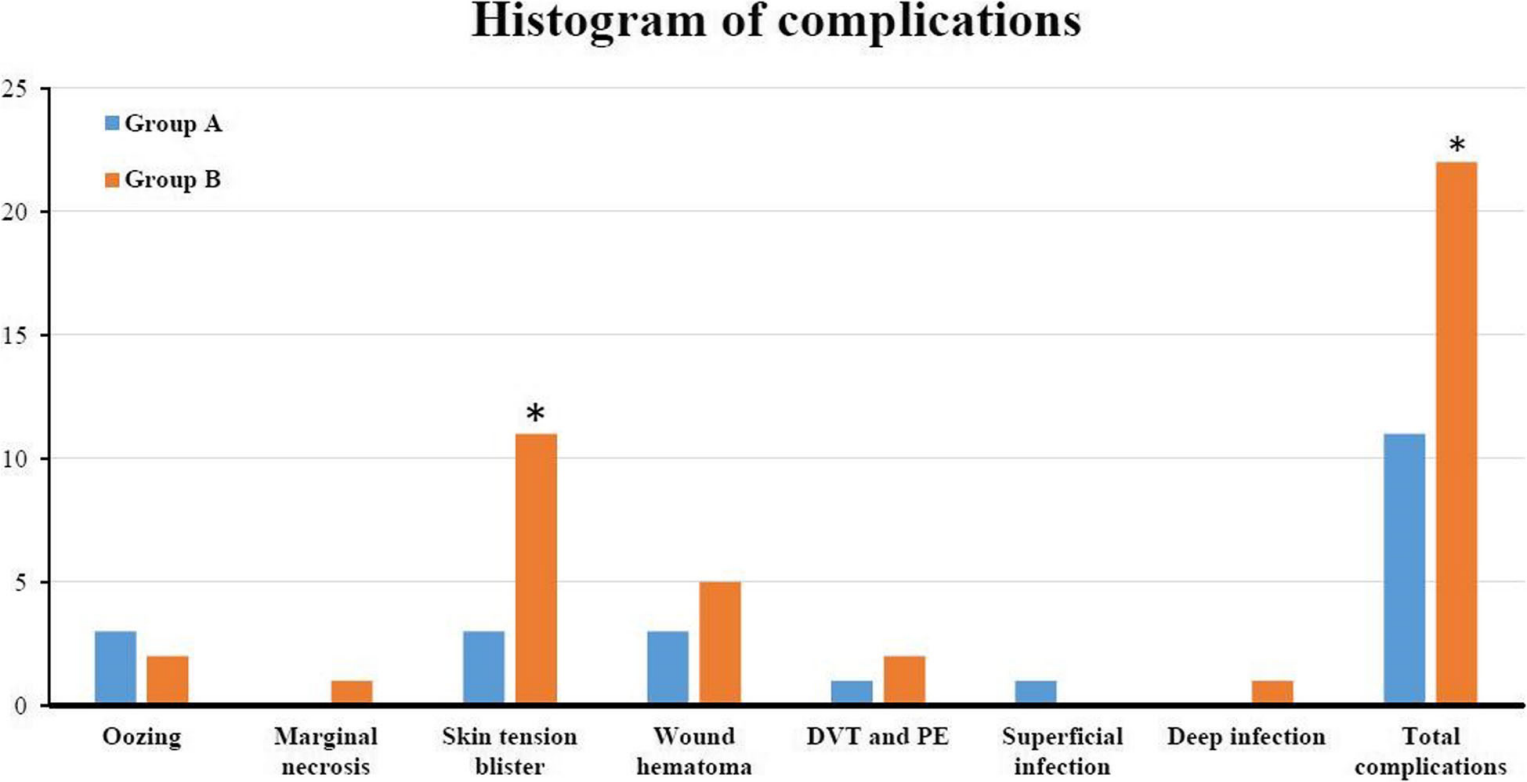
The histogram shows the complications between the groups, * indicates a significant difference

Significant difference was found on the complication of postoperative skin tension blister, three cases (2.5%) in Group A and eleven cases (9.3%) in Group B, (*P* = 0.031). Three cases of wound oozing, three cases of wound hematoma, one case of DVT and one case of superficial infection occurred in Group A, constituting a total of eleven (9.3%) complications; two cases of wound oozing, one case of incision marginal necrosis, five cases of wound hematoma, one case of DVT and one case of PE, one case of deep infection happened in Group B, constituting a total of 22 (18.6%) complications. Except for skin tension blister, no significant differences were found on other complications. Significant difference of total complications was found, however, no significant difference was found when excluding skin tension blisters (Figs. 4 and 5).

## Discussion

The most important findings of the present study were that the lower limb lifting is a safe and effective technique. Compared with squeeze exsanguination, it could decrease the incidence of skin tension blister, and alleviate early postoperative pain reaction. No additional risks presented regarding other clinical outcomes. Thus, it may potentially become a common exsanguination method during TKA surgery.

Skin tension blister, defined as the development of a fluid-filled vesicles under the epidermis, occurs when the epidermis is separated from the dermis and results from continuous friction on the skin, which has been a main problem with surgical wound following total joint arthroplasty(TJA) [28, 29]. It has been reported with an incidence of up to 20% after TJA [30]. The development of the postoperative skin tension blister can cause pain, discomfort and possible risk of superficial or even deep infections [31, 32]. Skin tension blister has been associated with variety factors, of which the most related factor is the choice of dressing as reported [28, 30]. Interestingly, a recent prospective study was conducted on 135 consecutive TKAs compared with a historical randomized controlled of 200 TKAs. Tourniquet was released immediately after wound closure to allow for re-perfusion and then a dressing was applied. It concluded that tourniquet release prior to dressing application could reduce the incidence of blister following TKA. As tourniquet release can result in a limb volume increase of 10%, they considered this benefit to the reduced friction between skin and dressing in the early tourniquet release randomized controlled [11]. In our study, the incidence of the skin tension blister was similar to this trial. Amazingly, we found a significant low incidence of skin tension blister in the lower limb lifting group. We ascribed this to three points: first, the hand-over-hand squeeze exsanguination might bring friction between gauze and skin when performing the squeeze; second, the soft tissue might be injured by the squeeze; and last, the preoperative increased level of ischemia might aggravate the injury of ischemia re-perfusion.

The most concerned questions are pain relief and functional recovery after TKA, which are main factors contributing to the satisfaction of patients. In the present study, another important interesting finding was the significant decrease of VAS score in the lower limb lifting group. Tanpowpong et.al [17] compared the effect of tight elastic bandaging and limb lifting exsanguination on upper extremity in 23 healthy adult volunteers. It demonstrated that the tourniquet tolerance was significantly lower in bandaging group. Farbood et.al [16] compared the esmarch bandage and limb lifting exsanguination during repairing upper extremity soft tissue injuries. Although they didn’t find significant statistical difference between pain data, the number of patients with more pain feeling were shown in the esmarch bandage exsanguination group. In the present study, we found a less than 0.5 VAS score increase in the squeeze group. We considered the reasons of increased soft tissue and ischemia re-perfusion injury by the preoperative squeeze.

Several limitations of this study should be noted here. Due to low incidence of some complications, the small sample size may not be enough; the senior surgeon could not be blinded to allocation, which may bring potential bias to the study; third, only a year’s follow-up outcomes were presented, long-term results such as rate of loosening or revision are anticipated; forth, the occurrence of DVT was determined by clinical symptoms instead of common ultrasound examination, which might have been underestimated. Though limitations exist, we conducted a prospective randomized controlled study and provided evidences supporting the lower limb lifting technique. We look forward to more related studies to further prove our claim.

## Conclusion

The lower limb lifting technique is a safe and effective technique. Compared to hand-over-hand squeeze exsanguination, it could decrease the incidence of skin tension blister, and alleviate early postoperative pain reaction. No additional risks presented regarding other clinical outcomes. Thus, it may potentially become a common technique during TKA procedure.

## Abbreviations

DVT: Deep vein thrombosis
HSS: Hospital for special surgery
MCS: Mental component summary
PCS: Physical component summary
PE: Pulmonary embolism
ROM: Range of motion
SF-36: 36-item short form surgery instrument
TJA: Total joint arthroplasty
TKA: Total knee arthroplasty
VAS: Visual analogue scale
